# Spontaneous meningioma in a pig-tailed macaque (*Macaca nemestrina*)

**DOI:** 10.5194/pb-5-7-2018

**Published:** 2018-04-05

**Authors:** Roland Plesker, Martina Bleyer, Kerstin Mätz-Rensing

**Affiliations:** 1 Paul-Ehrlich-Institut, Langen, 63225, Germany; 2 Pathology Unit, German Primate Center, Göttingen, 37077, Germany

## Abstract

We present a case of spontaneous meningioma in a female pig-tailed macaque (*Macaca nemestrina*) more than 24 years old. Clinically, the monkey displayed slow, weak, and insecure movements and poor vision. A tumorous mass was present at the floor of the cranial vault extending from the optic chiasm towards the foramen magnum. It compressed adjacent parts of the brain, infiltrated the sphenoidal and occipital bone, and showed transcranial expansion into the pharyngeal area. Histologically, the tumor was consistent with a meningioma displaying mostly meningothelial and some microcystic components. Since only six cases of meningiomas in nonhuman primates have been reported so far and only two of these meningiomas have been described in detail, the findings of each case should be reported to expand the knowledge base of this type of tumor. In addition, this is the first description of a meningioma in pig-tailed macaques.

## 1 Introduction

In humans, about 20 % of all primary intracranial tumors are meningiomas (Louis et al., 2000) and about 80–90 % of these are regarded as benign lesions (Whittle et al., 2004; Goldstein and Harsh, 2005; Harter et al., 2017). Meningioma most commonly develops in older people, and women are more often affected than men (Fonkem et al., 2016; Kalamarides and Goutagny, 2006; Louis et al., 2000; Longstreth Jr. et al., 1993; Wiemels et al., 2010; Whittle et al., 2004; Perry, 2006). In humans, the tumor is generally tightly attached to the dura mater (Nagashima et al., 2006). It often shows a slow expansive growth with compression of adjacent brain tissue and grows along the extensions of the dura mater (Whittle et al., 2004). The tumors are often well demarcated but might also show infiltrative growth (Perry et al., 1999).

Meningiomas are relatively common in cats (Zaki and Hurvitz, 1976) and dogs (Zaki and Hurvitz, 1976). In cats, 59 % of all intracranial tumors are meningiomas (Troxel et al., 2003) and 45 % of all intracranial tumors are meningiomas in dogs (Snyder et al., 2006). However, meningiomas are rare in cattle, sheep, and horses (Cantile and Youssef, 2016; Koestner and Higgins, 2002; Summers et al., 1995). Meningiomas are also known to occur in laboratory animals such as rats (Mitsumori et al., 1987) and mice (Summers et al., 1995).

Few studies have reported findings of meningiomas in nonhuman primates (Lowenstine, 1986; McClure, 1980). In prosimians, Winkelmann et al. (2007) reported a psammomatous meningioma in a black-and-white-ruffed lemur (*Varecia variegata variegata*), and Remick et al. (2009) documented a case of an anaplastic meningioma in a collared brown lemur (*Eulemur collaris*). In monkeys, Jungherr (1963) summarized necropsy results of 12 000 cynomolgus/rhesus monkeys, and observed one case of meningiomatosis in the lumbar cord. McConnell et al. (1974) briefly mentioned a meningioma in a survey of free-living chacma baboons in South Africa (*Papio ursinus*). In 2011, Oliveira et al. described an intracranial meningioma in a baboon (*Papio* spp.). Tanaka and Canfield (2012) published a case report of an intracranial meningioma with ophthalmoplegia in a rhesus macaque (*Macaca mulatta*).

Since reports of meningiomas in nonhuman primates are rare in the literature, we describe a case of a spontaneous meningioma in an aged pig-tailed macaque in this report. Histologically, the tumor displayed features of both meningothelial meningioma and of microcystic meningioma.

## 2 Animal and methods

### 2.1 Animal provenance

The affected animal was a female pig-tailed macaque (*Macaca nemestrina*) of at least 24 years of age. The exact date of birth was not documented. The pig-tailed macaque was obtained from a breeding colony in Slovenia and arrived at the German Primate Center in Göttingen, Germany, in 1993. In 1995, it was transferred to the Paul-Ehrlich-Institut (PEI) in Langen, Germany, where it lived for 21 years in an experimental indoor facility. It was group- or pair-housed in accordance with European and German animal welfare legislation and produced seven offspring. The monkey was used for experimental blood collection.

### 2.2 Housing

The cage was made of steel with a size of 300 cm × 375 cm × 225 cm. Large windows allowed the monkey to watch the outside environment. Natural branches, ropes, nets, bedding, mirrors, kong toys, puzzle feeders, prima-hedrons, music, and television were supplied for environmental enrichment. The diet consisted of monkey pellets ad libitum (Trio Munch^®^, Special Diet Services/Mazuri, Witham, England) in the morning and seasonal vegetables and fruits twice weekly. The monkey was also offered a mixture of nuts, mealworms, rice, popcorn, and curd.

### 2.3 Clinical history

The monkey had acquired multiple bite injuries on different parts of its body during its time in the PEI group housing facility. It additionally had slow, insecure, and weak movements, and its vision had deteriorated progressively over the past 3 years. The toes of the left foot had been kept in a rigid claw-like grasping position for at least 5 years. Over the past 3 years, the animal developed two slowly growing subcutaneous tumors with a size of 4×3 cm each at the ventral abdomen close to the linea alba. A general atrophy of both the epaxial and the appendicular muscles became obvious during the last year before its death.

The proximal cause for the euthanasia of the animal was a combination of a laceration of the skin and muscle on the left arm and pain vocalization during walking and climbing movements within the cage.

The animal was euthanized by intravenous injection of T 61 (Intervet Deutschland GmbH, Unterschleißheim, Germany) under deep ketamine–xylazine anesthesia (Ketamin 10 %, WDT, Garbsen, Germany; Rompun^®^, Bayer Vital GmbH, Leverkusen).

Necropsy was performed immediately after euthanasia. Photographs were taken and organs of interest were fixed in 4 % formaldehyde solution for 7 days before processing. Paraffin embedding of fixed tissues, preparation of 4 µm sections, and hematoxylin–eosin staining were carried out in accordance with standard procedures (Mulish and Welsch, 2015). Bones were decalcified with 5–15 % hydrogen chloride (Decal^®^, SERVA Electrophoresis GmbH, Heidelberg, Germany) for the production of histological slides according to manufacturer's instructions.

### 2.4 Antibodies

Immunohistochemical examinations were performed on paraffin-embedded sections using the following primary antibodies commercially available from DakoCytomation GmbH, Hamburg, Germany: anti-Ki67 antibody (monoclonal mouse anti-human Ki67 antigen, clone MIB-1, 1:50), anti-vimentin antibody (monoclonal mouse anti-human vimentin antigen, clone V9, 1:100), anti-cytokeratin antibody (monoclonal mouse anti-human multi-cytokeratin, clone MNF116, 1:100), anti-GFAP antibody (polyclonal rabbit anti-human glial fibrillary acidic protein, 1:500), anti-S100 antibody (polyclonal rabbit anti-human S100A1, 1:1000), anti-NSE antibody (monoclonal mouse anti-human neuron specific enolase, clone BBS/NC/VI-H14, 1:400), and anti-SMA antibody (monoclonal mouse anti-human smooth muscle actin, clone 1A4, 1:400). Immunohistochemistry was performed in an automated immunostaining system (Discovery XT, Roche Diagnostics GmbH, Mannheim, Germany) using the SABC (streptavidin–biotin complex) method and DAB (diaminobenzidine tetrahydrochloride) for signal detection (DAB Map Kit, Roche Diagnostics GmbH, Mannheim, Germany). All primary antibodies used in this case report have previously been validated and successfully used in rhesus macaques, a closely related macaque species (Gruber-Dujardin et al., 2017; Vogel and Fritz, 2003). Corresponding tissue sections from rhesus macaques were used as positive controls to demonstrate antibody specificity. Pure antibody diluent instead of primary antibody was applied to the negative control sections to visualize possible nonspecific binding of the secondary antibody. Immunohistochemical staining for epithelial membrane antigen (EMA) was performed using the EnVision Detection System (Agilent/Dako, Denmark) and the commercially available monoclonal antibody EMA (Clone E29, Ready-to-Use) was employed. Samples were visualized with the EnVision FLEX System (Autostainer Link 48, Agilent/Dako, Denmark).

## 3 Results

**Figure 1:**
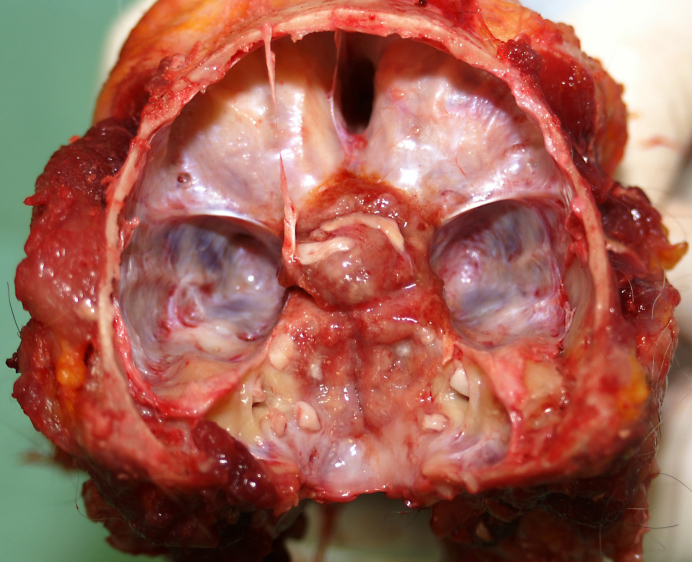
View of the cranial base of an old pig-tailed macaque (brain removed): the meningioma was located around the hypophyseal stalk involving the optic chiasm.

At necropsy, a tumorous mass was detected at the base of the ossified cranium after the brain was removed. It was centered around the hypophyseal stalk, extending cranially toward the optic chiasm, and a thin tumor tissue layer extended caudally towards the foramen magnum (Fig. 1). The tumor was well vascularized and primarily light red or beige in color, although some areas had light grey elements. It had an elastic consistency, and some regions were slightly edematous. Its surface was mainly smooth, but revealed a slightly rough surface in the thinner caudal parts of the tumor. The tumor was firmly attached to the dura mater, well demarcated, and did not invade the brain macroscopically. The spinal cord was not examined. The paranasal sinuses showed no abnormalities.

**Figure 2:**
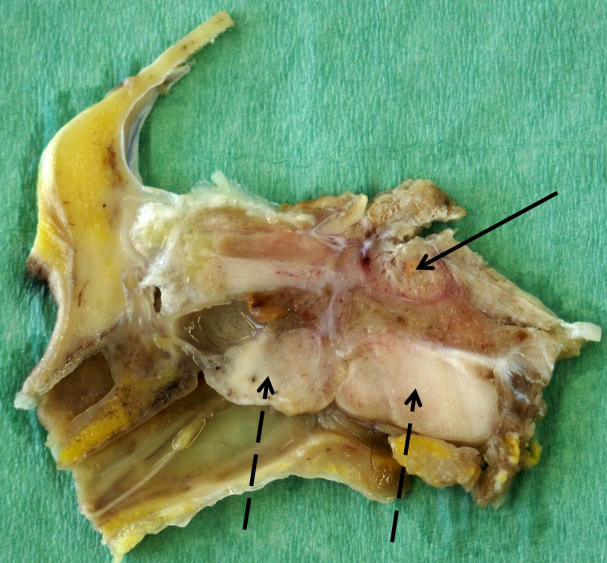
Median cross section through the formalin-fixed skull of an old pig-tailed macaque (brain and tip of the nose removed): meningioma in the hypophyseal fossa (black arrow) and in the pharyngeal area (dashed arrows).

In the skull cross section, the hypophyseal fossa was completely filled with tumor tissue, which was demarcated by a red margin from surrounding bones. The cross section revealed two additional firm white parts of the tumor (2 cm × 1.5 cm and 3 cm × 1.5 cm) located in the median between the epithelium of the pharynx and parts of the sphenoidal and occipital bones, compressing both the pharyngeal and the esophageal lumen (Fig. 2).

The two oval tumors on the ventral abdominal wall were identified as lipomas. Several joints displayed arthrosis of the cartilage. In addition, spondylosis was detected in the thoracic and lumbar portion of the spinal column. A slight scoliosis was also present in the thoracic area. In the right ovary, a 0.5 cm diameter large thin-walled cyst was evident containing clear watery fluid.

The intracranial tumor completely filled the space around the pituitary gland (fossa hypophysealis). However, there was no infiltration into the pituitary gland. In contrast, there was extensive invasion into surrounding bones. Tumor cells were also attached to the perineurium of the optic nerve at the connection to the eye.

**Figure 3:**
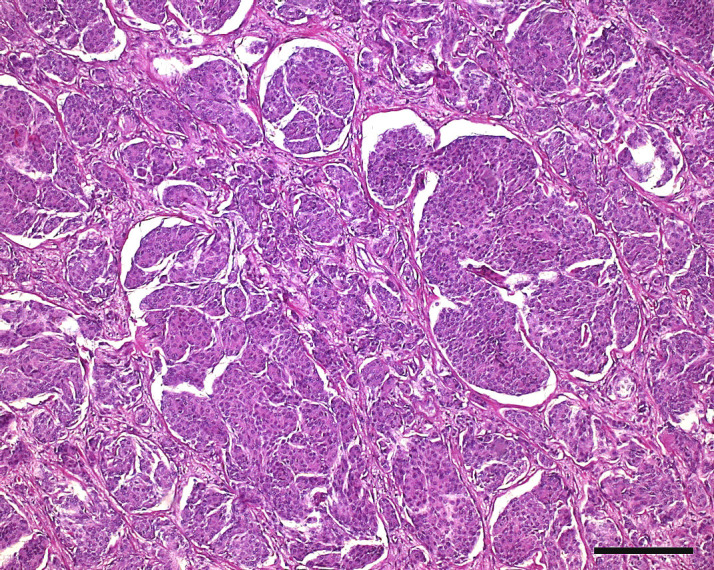
Histological photograph of a meningioma in an old pig-tailed macaque: meningothelial portion of the meningioma with islands of tumor cells separated by thin fibrous stroma. (hematoxylin–eosin, scale bar = 200 µm)

The tumor displayed two main histological cell types. In subepithelial areas of the pharynx and around the pituitary gland, the tumor consisted of multiple ovoid islands or nests of tumor cells separated by fine junctions of fibrous tissue (Fig. 3). Occasionally, cells were arranged in indistinct whorls. The islands consisted of numerous small polygonal cells with indistinct cell borders and with moderate amounts of eosinophilic cytoplasm. Nuclei were uniform, round to ovoid, and condensed with finely stippled nuclear chromatin. Only one nucleolus was normally visible. There was mild anisokaryosis and anisocytosis, and mitotic figures were rarely observed. Overall, an island-like hepatoid appearance of the tumor with partly whorl-like layers of cells was the prominent histological characteristic consistent with human World Health Organization (WHO) grade I meningothelial meningioma. Within these areas, the tumor produced few small spots of dystrophic lamellar calcification (psammoma bodies) and very few areas with regional mucin production. This histological appearance occurred in about 65 % of the tumor mass.

**Figure 4:**
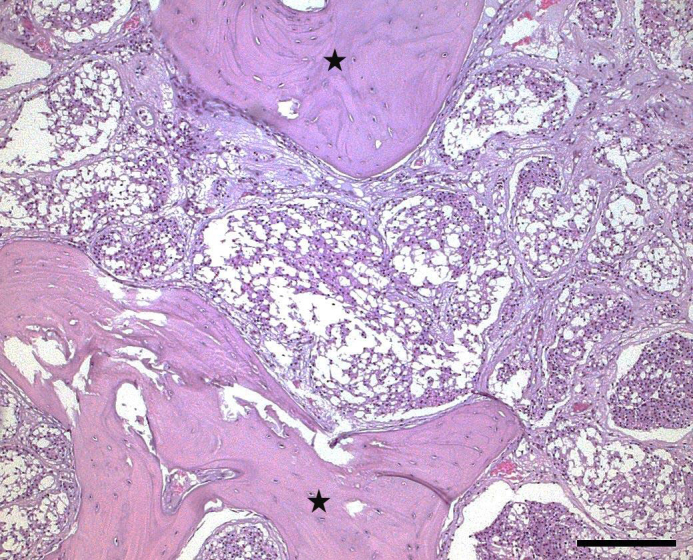
Histological photograph of a meningioma in an old pig-tailed macaque: Microcystic part of the meningioma characterized by vacuolated tumor cells. (hematoxylin–eosin, scale bar = 200 µm; asterisks: bones)

Tumor cells in surrounding bones had larger amounts of vacuolated, apparently empty pale cytoplasm and smaller, more condensed nuclei (Fig. 4). These cells also exhibited mild anisokaryosis and anisocytosis, while mitoses were infrequent. The separating fibrous tissue was also vacuolated. This histological appearance is consistent with human WHO grade I microcystic meningioma and was evident in approximately 35 % of the tumor mass.

The tumor showed immunoreactivity for vimentin (100 % of tumor cells) and very few tumor cells stained positive for Ki67. However, the tumor was negative for cytokeratin, S 100, glial fibrillary acidic protein (GFAP), neuron-specific enolase (NSE), smooth muscle actin (SMA), and EMA, while positive controls demonstrated the specificity of the antibodies.

## 4 Discussion

Clinical signs of meningiomas are normally the result of the compression of neighboring structures, and are therefore dependent upon tumor location (Perry, 2006; Summers et al., 1995; Whittle et al., 2004). Common clinical signs in dogs and cats are altered consciousness, seizures, and vestibular dysfunction (Motta et al., 1987). In this case, the monkey had a history of poor vision and slow, insecure, and weak movements. While these manifestations could have different causes (for example, joint alterations as the cause of slow movements), it cannot be excluded that they may be caused by the meningioma. In this context, visual impairment was reported as one clinical sign in a baboon with meningioma (Oliveira et al., 2011). In addition, eye muscles were affected in a rhesus macaque with a meningioma (Tanaka and Canfield, 2012). It is noteworthy that in our case no histological changes were detected within the eyes. However, parts of the meningioma in our case were evident close to the optic nerve, a condition that is reported in domestic animals as well (Koestner et al., 1999), which can also cause visual impairment in humans (Li et al., 2017). While further histological investigation of the nerve was not conducted, an influence of the tumor on the vision cannot be completely excluded.

Meningiomas originate from the arachnoid (Kepes, 1986) or meningeal progenitor cells (Kalamarides et al., 2011) and are normally firmly attached to the meninges. They can occur anywhere along the meninges, including the optic nerve and spinal cord. Meningiomas in humans are commonly reported in the skull vault, the skull base, sites of dural reflections, and less commonly in the optic nerve sheath and the choroid plexus. Approximately 10 % of human meningiomas arise in the spine (Whittle et al., 2004). In dogs, the olfactory bulb, frontal lobes, the floor of the cranial cavity, the optic chiasm, and the suprasellar or parasellar regions are commonly affected (Patnaik et al., 1986; Snyder et al., 2006; Sturges et al., 2008). In cats, the tela choroidea of the third ventricle and dorsal and lateral convexities are involved (Koestner et al., 1999; Troxel et al., 2003). In the present case of the pig-tailed macaque, the tumor was found at the base of the ossified cranium as previously described for a baboon (Oliveira et al., 2011).

Meningiomas are typically considered to be benign tumors and are normally well-demarcated masses of soft to firm consistency (Summers et al., 1995; Whittle et al., 2004). Meningiomas normally do not invade the brain but they compress neighboring structures (Frankhauser et al., 1974). However, in this case, the meningioma showed infiltrative growth into surrounding bones, which sometimes occurs in humans (Scott, 1992; Spille et al., 2016).

Canine and human meningiomas are currently histologically classified according to human WHO criteria (Louis et al., 2016), but this grading system is actually not applicable to feline meningiomas (Mandara et al., 2010). WHO classification categorizes meningiomas into 15 variants: grade I (benign, nine variants), grade II (intermediate, three variants), and grade III (malignant, three variants) according to their morphological and biological behavior (Table 1).

**Table 1:** The WHO classification of meningiomas (modified).

Grading	Subtypes
Grade I (benign)	meningothelial fibrous transitionalpsammomatous angiomatous microcystic secretory lymphoplasmacyte-richmetaplastic
Grade II Mitotic index 4–19 per 10 HPF, brain invasion possible	chordoid clear cell or atypical with three or more of the following criteria: increased cellularity, small cells with high nuclear-to-cytoplasmic ratio, prominent nucleoli, sheeting, foci of spontaneous necrosis
Grade III (malignant) Mitotic index ≥20 per 10 HPF	papillary rhabdoid or anaplastic (malignant) with overtly malignant cytology: high-grade sarcoma-, carcinoma-, or melanoma-like appearance, markedly elevated mitotic activity, often extensive necrosis, and a Ki67 proliferation index ≥20 %

Different subtypes may occur within one meningioma.

Whereas human meningiomas are histologically classified as, for example, 94.2 % for grade I, 4.2 % for grade II, and 1.57 % for grade III (Dolecek et al., 2015, for the US in 2004–2011), canine meningiomas are histologically classified as 56 % for grade I, 43 % for grade II, and 1 % for grade III (Sturges et al., 2008). In contrast, grade III meningiomas were not detected in cats (Mandara et al., 2010). Generally, metastases of meningiomas are rare in humans (Enam et al., 2005), dogs (Motta et al., 1987; Pérez et al., 2005), and cats (Dahme, 1957; Motta et al., 1987). Histologically, most meningiomas do not exhibit cellular criteria of malignancy. In our case, the cells were well differentiated and displayed only a few mitoses, which is largely consistent with benign human meningiomas (0.08±0.05 mitoses per 10 HPF for benign, 4.75±0.91 mitoses per HPF for atypical, and 19.00±4.07 mitoses per HPF for malignant) (Hsu et al., 1994).

Some authors discuss advantages of the human system of classification compared with the current WHO classification for animals (Koestner et al., 1999; Mandara et al., 2010; Sturges et al., 2008). Due to these considerations and due to the evolutionary relatedness between nonhuman primates and humans, we referred to the human WHO classification in order to classify the tumor in this case. According to this classification, we diagnosed a meningioma that showed histological appearance both of a meningothelial meningioma (65 % of the tumor mass) and a microcystic meningioma (35 % of the tumor mass).

Meningiomas do not have definitive cytologic markers, and the pathologic diagnosis is usually made on the basis of tumor cytoarchitecture (Louis et al., 2016). In humans, EMA and vimentin are usually the most reliable immunohistochemical markers (Pérez-Guiones Bacete et al., 1992; Schnitt and Vogel, 1986; Schwechheimer et al., 1984; Winek et al., 1989), although many tumors are also positive for cytokeratin (Pérez-Guiones Bacete et al., 1992; Perry, 2006; Winek et al., 1989). S-100 protein immunostaining is variable (Pérez-Guiones Bacete et al., 1992; Schnitt and Vogel, 1986; Winek et al., 1989) and GFAP expression is rare (Wanschitz et al., 1995). In humans and domestic carnivores, the MIB-1 antibody against the Ki67 antigen was successfully correlated with the histological grade of meningeal neoplastic cells (Devaprasath and Chack; 2003; Maes et al., 2005; Mandara et al., 2002). In this case, the tumor only showed immunoreactivity for vimentin (100 % of tumor cells) and a few tumor cells were positive for Ki67. However, it was negative for cytokeratin, S 100, GFAP, NSE, SMA, and EMA, which is consistent with what has been reported in other studies. However, it cannot be excluded that some of the negative staining results are false negative in the present case, which might be attributed to antigen impairment during the decalcification process prior to embedding. Lack of antibody cross reactivity with pig-tailed macaque tissue seems unlikely in view of the positive reaction with rhesus macaque tissue. However, the significance of the immunohistochemical results remains questionable in the present case and the diagnosis of meningioma mainly relies on the histological appearance of the tumor.

## 5 Conclusions

This report is the first description of a meningioma in a pig-tailed macaque (*Macaca nemestrina*) and one of only three detailed descriptions of meningiomas in nonhuman primates in the literature, and contributes to the knowledge of this tumor entity in nonhuman primates.

## Data Availability

Paraffin-embedded organ material is available via the corresponding author.
